# The Effect of Exogenous Ketone Monoester Ingestion on Plasma BDNF During an Oral Glucose Tolerance Test

**DOI:** 10.3389/fphys.2020.01094

**Published:** 2020-09-09

**Authors:** Jeremy J. Walsh, Étienne Myette-Côté, Jonathan P. Little

**Affiliations:** ^1^School of Health and Exercise Sciences, University of British Columbia Okanagan, Kelowna, BC, Canada; ^2^Department of Kinesiology, McMaster University, Hamilton, ON, Canada; ^3^Department of Medicine, Université de Sherbrooke, Sherbrooke, QC, Canada; ^4^Research Center on Aging, Université de Sherbrooke, Sherbrooke, QC, Canada

**Keywords:** β-hydroxybutyrate, adults with obesity, lean adults, brain-derived neurotrophic factor, glucose-lowering, metabolism, placebo-controlled, double-blind

## Abstract

Brain-derived neurotrophic factor (BDNF) is important for brain and metabolic function. Ingestion of a ketone monoester (KME) drink containing beta-hydroxybutyrate (β-OHB) attenuates hyperglycemia in humans and increases neuronal BDNF in rodents. Whether KME affects BDNF in humans is currently unknown. This study examined the effect of KME ingestion before an oral glucose tolerance test (OGTT) on plasma BDNF in normal-weight adults (NW) and adults with obesity (OB).

**Methods**: Exploratory, secondary analyses of two studies were performed. Study 1 included NW (*n* = 18; age = 25.3 ± 4.3 years; BMI = 22.2 ± 2.3 kg/m^2^) and Study 2 included OB (*n* = 12; age = 48.8 ± 9.5 years; BMI = 33.7 ± 5.0 kg/m^2^). Participants ingested 0.45 ml/kg^−1^ body weight KME or Placebo 30-min prior to completing a 75 g OGTT. β-OHB and BDNF were measured *via* blood samples at fasting baseline (pre-OGTT) and 120 min post-OGTT.

**Results**: Study 1: KME significantly increased β-OHB by 800 ± 454% (*p* < 0.001). BDNF significantly decreased post-OGTT compared to pre-OGTT in Placebo (718.6 ± 830.8 pg/ml vs. 389.3 ± 595.8 pg/ml; *p* = 0.018), whereas BDNF was unchanged in KME (560.2 ± 689.6 pg/ml vs. 469.2 ± 791.8 pg/ml; *p* = 0.28). Study 2: KME significantly increased β-OHB by 1,586 ± 602% (*p* < 0.001). BDNF was significantly higher post-OGTT in the KME condition in OB (time × condition interaction; *p* = 0.037). There was a moderate relationship between β-OHB and ∆ %BDNF (r = 0.616; *p* < 0.001). Fasting plasma BDNF was significantly lower in OB compared to NW (132.8 ± 142.8 pg/ml vs. 639.4 ± 756.8 pg/ml; *g* = 0.845; *p* = 0.002).

**Conclusions**: Plasma BDNF appears differentially impacted by KME ingestion with OGTT in OB compared to NW. Raising β-OHB *via* KME may be a strategy for increasing/protecting BDNF during hyperglycemia.

## Introduction

Brain-derived neurotrophic factor (BDNF) is a neurotrophin that exerts considerable regulatory effects on brain plasticity and whole-body energy homeostasis (Marosi and Mattson, 2014). BDNF promotes the growth and survival of central and peripheral neurons and protects against stress-induced apoptosis (Almeida et al., 2005). BDNF also exerts metabotropic effects in the arcuate nucleus of the hypothalamus by impacting long-term feeding behaviors (Kernie, 2000; Marosi and Mattson, 2014). A variety of *bdnf* knockout or BDNF blockade studies support the importance of BDNF for optimal neural and metabolic health (for extensive reviews on these pleiotropic effects, cf.: Marosi and Mattson, 2014).

Cross-sectional evidence in humans shows a negative gradient relationship between body mass index (BMI), glycemic control, and plasma BDNF such that lean, normoglycemic individuals had the highest plasma BDNF and people with obesity and type 2 diabetes (T2D) had the lowest (Krabbe et al., 2007). BDNF expression is sensitive to energy status, and energetic stressors like exercise and fasting upregulate BDNF expression (Walsh et al., 2015; Dinoff et al., 2017). Conversely, acute hyperglycemia reduces cerebral output of BDNF in young adults, which may be a mechanism to explain lower plasma BDNF in adults with T2D (Krabbe et al., 2007). Impaired glucose tolerance is associated with accelerated brain atrophy, impaired cognitive function, and increased risk of neurological disorders, which may in part be due to reductions in BDNF (Cherbuin and Walsh, 2019). Strategies that can attenuate hyperglycemia may positively impact circulating BDNF.

Recently, we demonstrated that ingestion of a ketone monoester (KME), an (R)-3-hydroxybutyl (R)-3-hydroxybutyrate drink, attenuates the glycemic response to an oral-glucose tolerance test (OGTT) by 16% in lean adults (Myette-Côté et al., 2018) and 11% in adults with obesity (Myette-Côté et al., 2019). In rodents, beta-hydroxybutyrate (β-OHB) has been shown to directly upregulate BDNF expression in cultured cortical and hippocampal neurons but its effect in humans remains unknown (Marosi et al., 2016; Sleiman et al., 2016; Hu et al., 2018). Taken together, the ability of β-OHB to attenuate hyperglycemia along with its potential to upregulate neuronal BDNF expression presents as a novel strategy for improving metabolic and brain health in people with increased cardiometabolic risk. Whether BDNF is impacted by ingestion of β-OHB during an OGTT is currently unknown. Therefore, the purpose of this report was to examine the effect of KME ingestion before an OGTT on plasma BDNF in normal-weight (NW) adults and adults with obesity (OB). The present report represents secondary data analysis from two separate published studies (Myette-Côté et al., 2018, 2019).

## Materials and Methods

Herein, we present secondary data analyzed from two previously published studies (Myette-Côté et al., 2018, 2019) and a previously published conference abstract (Walsh et al., 2020). Briefly, a double-blind placebo-controlled crossover design was used to investigate the effect of KME ingestion 30 min prior to a 75-g OGTT on metabolic control in NW (Myette-Côté et al., 2018) and OB adults (Myette-Côté et al., 2019). Two-hour glucose area under the-curve (AUC) was the primary outcome of these studies and serial blood samples were collected to assess other hormones and metabolites (e.g., insulin, c-peptide, and non-esterified fatty acids) as secondary outcomes. The plasma samples used in the current analysis were never previously thawed and were kept frozen at −80°C for less than 2 years for potential future secondary analyses. Although the populations differed, the same methodology was used for both studies.

### Participants

#### Study 1

Twenty young healthy adults aged between 18 and 35 years participated to this study (Table 1). Among them, we were able to obtain plasma BDNF concentrations from 18 participant samples. Exclusion criteria included: (1) currently taking any medications (except for birth control for females), (2) following a low-carbohydrate diet or intermittent fasting protocol, (3) consuming nutritional ketone supplements, (4) being engaged in intensive exercise training (>300 min/week of planned physical activity), and (5) having waist circumference >102 or >88 cm for men and women, respectively.

**Table 1:** Participant characteristics.

	Study 1 (NW; *n* = 18)	Study 2 (OB; *n* = 12)
Male/female	9/9	3/9
Age (years)	25.3 (4.3)	48.8 (9.5)^*^
Body mass index (kg/m^2^)	22.2 (2.3)	33.7 (5.0)^*^
Waist circumference (cm)	70.5 (7.3)	103.1 (12.4)^*^
Systolic BP (mmHg)	116 (8)	128 (11)^*^
Diastolic BP (mmHg)	76 (8)	84 (8)^*^
Fasting glucose (mmol/L)^a^	4.5 (0.4)	5.8 (1.0)^*^

All values reflect only the participants analyzed in the current, secondary data analysis. BP, blood pressure; NW, normal weight adults; OB, adults with obesity.

a Glucose data are the average of two fasting measures made at baseline [pre-oral-glucose tolerance test (OGTT)] of each condition.

* Significantly different compared to NW, *p* < 0.05.

#### Study 2

Fifteen adults with obesity aged between 30 and 65 years participated to this study (Table 1). Among them, we were able to obtain BDNF concentrations from 12 participant samples. Inclusion criteria required participants to have a waist circumference >102 or >88 cm for males and females, respectively, combined with a body mass index ≥28 kg/m^2^. Exclusion criteria included: (1) taking medications affecting glucose or lipid metabolism, (2) following a low-carbohydrate diet or intermittent fasting protocol, (3) consuming ketone supplements, (4) engaging in intensive exercise training (>300 min/week of planned physical activity), and (5) being diagnosed with diabetes or heart disease. Baseline characteristics for the participants included in the current analysis can be found in Table 1.

### Experimental Procedures

#### Baseline Testing

Body weight (kg), height (cm), blood pressure (mmHg), and waist circumference (cm) were measured using standardized methods. A 24-h food log was provided to participants to ensure replication of meals consumed 24-h prior to each experimental visit. Participants were also instructed not to consume alcohol or to exercise for 24-h before each experimental trial.

#### Experimental Trial

Participants reported to the laboratory for experimental trials after an overnight fast (≥10 h). The research coordinator confirmed with them that the 24-h food log had been completed, that no exercise had been performed, and that no alcohol had been consumed on the day before. An indwelling intravenous catheter (BD Nexiva, Becton Dickinson Infusion Therapy Systems Inc., Utah, USA) was then inserted into the antecubital vein for repeated blood sampling. At each time point, blood was drawn into 1 × 2 ml EDTA (BD Vacutainer, Becton Dickinson Infusion Therapy Systems Inc., Utah, USA) for isolation of plasma. Seven intravenous blood draws were performed for each condition. The first collection (−30 min) was immediately followed by the consumption of the drink (placebo or KME). Participants consumed a KME supplement in the form of (*R*)-3-hydroxybutyl (*R*)-3-hydroxybutyrate (ΔG® from TΔS® Ltd., UK; 0.45 ml/kg body mass or 482 mg/kg body mass) ingested with water and calorie-free stevia (SweetLeaf) in a total volume of 100 ml (Study 1) or water and calorie-free artificial sweetener (Mio, Kraft Foods) in a total volume of 150 ml (Study 2). In the placebo condition, participants consumed 100 ml of water and calorie-free stevia (SweetLeaf; Study 1) or 140 ml of water combined with 10 ml of bitter flavor (Symrise, 6,48,352) and a calorie-free artificial sweetener (Mio, Kraft Foods). Participants wore a nose clip while consuming the drinks to further mask any flavor. Thirty minutes later, a second blood sample was collected (0 min) followed immediately by the consumption of a 75-g OGTT drink (Thermo Scientific, Fisher Scientific Company, Middletown, USA). Another five blood draws occurred at 15, 30, 60, 90, and 120 min after ingestion of the glucose drink. At each time point, β-OHB was measured in whole blood using β-ketone strips (Precision Neo, Abbott Laboratories, Witney, UK). For the current exploratory analyses, only blood samples at time 30 (Rest) and 120 min (post-OGTT) were used.

In a randomized crossover design, participants returned to the laboratory (≥10 h fast) at least 48 h later to complete the second condition with the alternate supplement. Participants and study personnel performing laboratory blood sample analyses were blinded to experimental conditions using coding (A for first and B for second visit). A third-party, unblinded researcher prepared all supplements and collected all blood samples.

### Blood Processing and Biochemical Analysis

Blood was drawn in EDTA tubes and centrifuged immediately at 1500 *g* at 4°C for 15 min. The resultant supernatant was aliquoted and stored at −80°C. Given that this is a post-hoc secondary, exploratory analysis, a second centrifuge spin to remove platelets was not performed.

Plasma BDNF was measured *via* a commercially available enzyme-linked immunoassay (BEK-2211-2P, Biosensis, SA, Australia). The intra‐ and inter-assay coefficients of variation are 1.0 and 5.0%, respectively, as reported by an independent third party (Polacchini et al., 2015). All steps were followed in accordance with the manufacturer’s instructions, with the exception of the recommended 10-fold dilution for plasma samples with higher BDNF concentrations. Our in-house assay optimization pilot testing revealed that 5-fold sample dilution was optimal to ensure that samples were within the 7.8–500 pg/ml standard curve detection range. Plasma BDNF represents the unbound, biologically available pool of circulating BDNF. Addition of BDNF into the plasma pool comes from numerous cellular sources and circulating platelets (Walsh and Tschakovsky, 2018).

### Statistical Analysis

Separate linear mixed-effects models were computed to assess the BDNF response to KME or placebo during an OGTT (time × condition) for NW and OB. Significant interactions were followed up with pairwise comparisons across time within conditions using a Tukey post-hoc test. An independent samples t-test was used to compare participant characteristics and fasting (pre-OGTT) plasma BDNF between lean and obese participants. Statistical significance was set at *p* < 0.05. Hedges’ g was used to evaluate effect sizes for pairwise comparisons indicated. All statistical analysis was performed using Prism 8 GraphPad (GraphPad Software, San Diego, USA).

## Results

### Study 1: The Effect of KME Ingestion With OGTT on Plasma BDNF in NW

**Figure 1:**
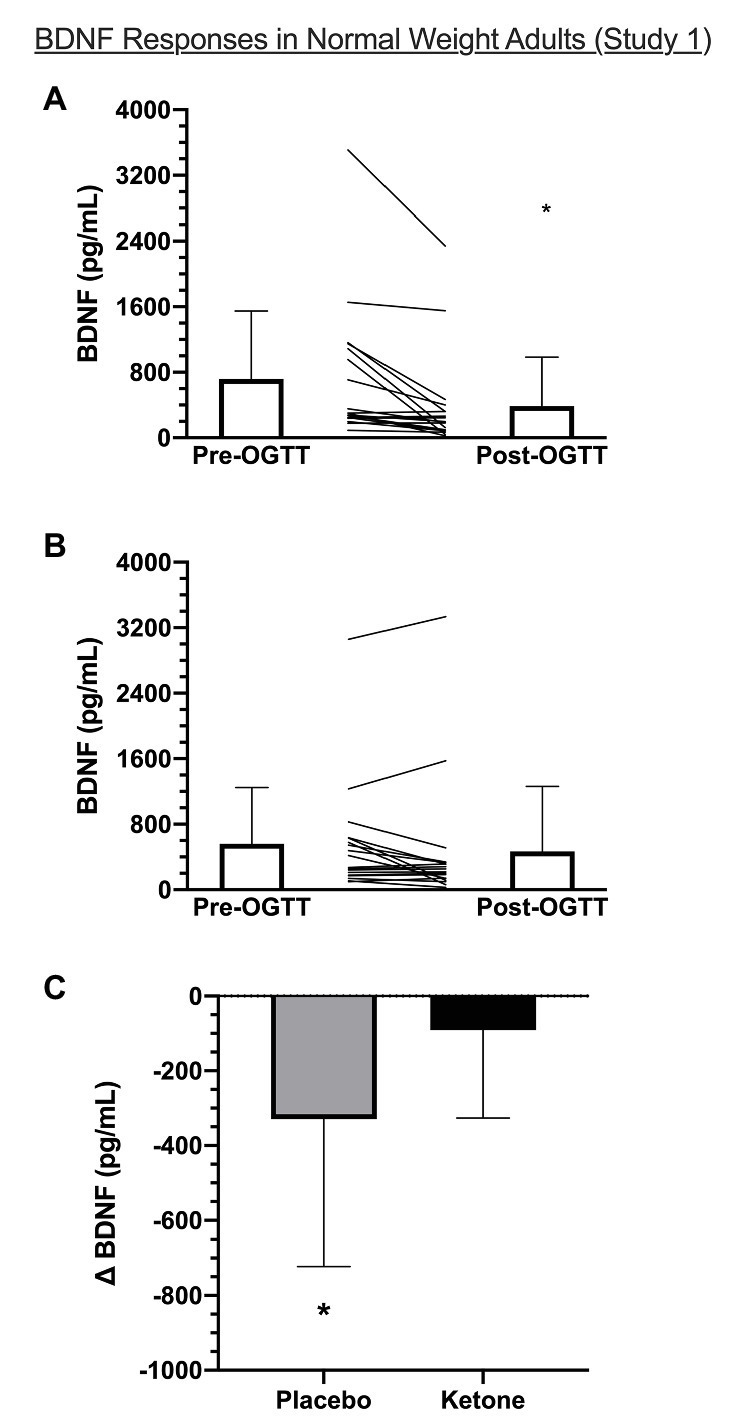
Plasma BDNF before (pre-OGTT) and 120 min after (post-OGTT) consumption of **(A)** placebo or **(B)** ketone + OGTT in NW (Study 1). **(C)** shows the change in brain-derived neurotrophic factor (BDNF; post-minus pre-OGTT) within each condition. Bar graphs are group mean ± SD and lines are individual BDNF responses for a given condition. ^*^Significantly different compared to Rest in the same condition, *p* < 0.05.

In the current analysis, plasma β-OHB increased from 0.18 ± 0.10 mM at fasting baseline (pre-OGTT) to 1.31 ± 0.34 mM at 120 min post-OGTT in the KME condition (*p* < 0.001). The corresponding AUC over the 120 min OGTT period for β-OHB was 276.1 ± 45.6 mM × 120 min in KME compared to 23.1 ± 3.8 mM × 120 min in the placebo condition (*p* < 0.001). The peak β-OHB concentration for NW was 3.2 ± 0.6 mM and occurred 15-min post-KME ingestion (see Myette-Côté et al., 2018). There was a significant time x condition interaction (*p* = 0.009) for the BDNF response to KME ingestion during the OGTT in NW (Figure 1). In the placebo condition, plasma BDNF was significantly decreased at the end of the OGTT compared to pre-OGTT (389.3 ± 595.8 pg/ml vs. 718.6 ± 830.8 pg/ml; *p* = 0.004), whereas in the KME condition, post-OGTT plasma BDNF was unchanged compared to pre-OGTT (560.2 ± 689.6 pg/ml vs. 469.2 ± 791.8 pg/ml; *p* = 0.608).

### Study 2: The Effect of KME Ingestion With OGTT on Plasma BDNF in OB

**Figure 2:**
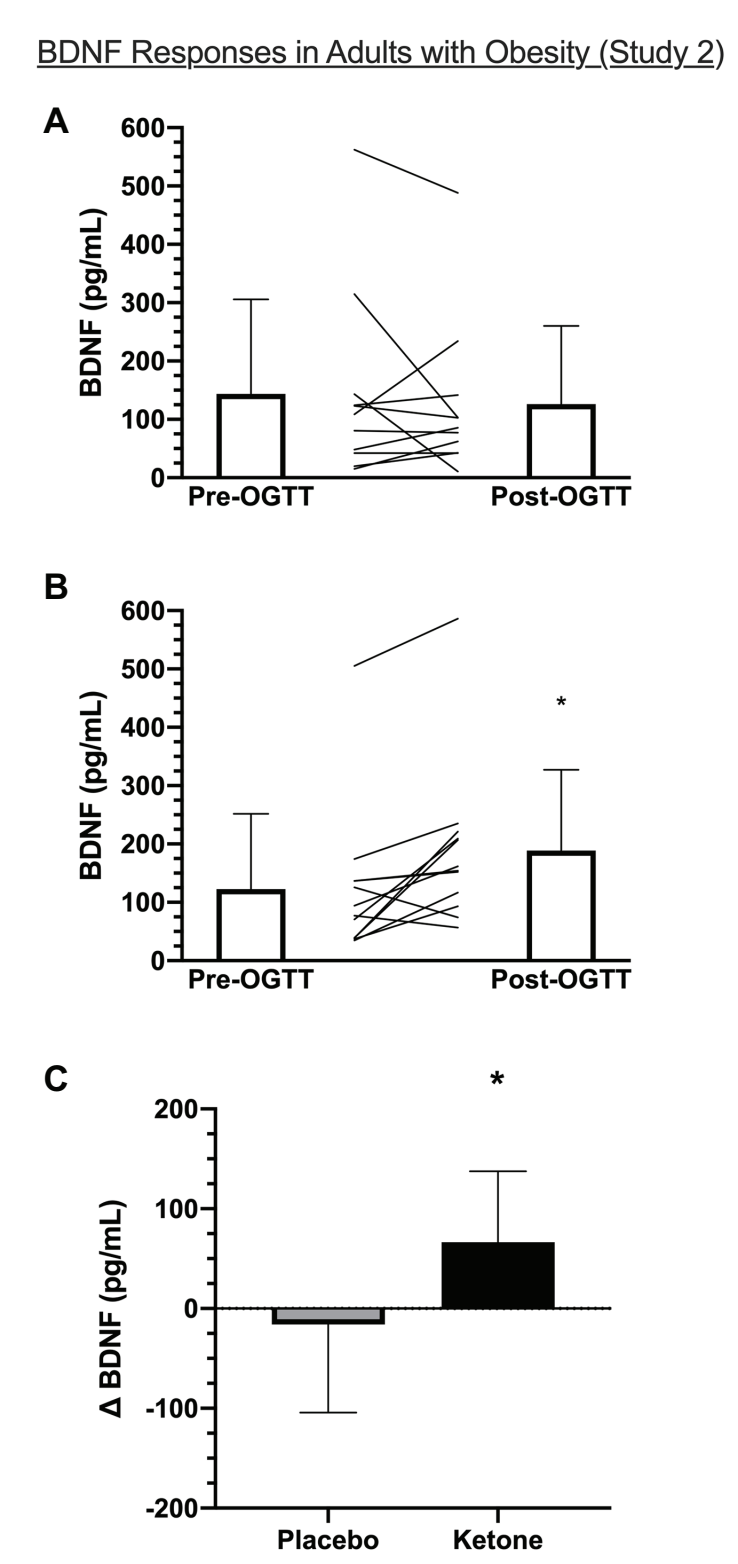
Plasma BDNF before (pre-OGTT) and 120 min after (post-OGTT) consumption of **(A)** placebo or **(B)** ketone + OGTT in OB (Study 2). **(C)** shows the change in BDNF (post-minus pre-OGTT) within each condition. Bar graphs are group mean ± SD and lines are individual BDNF responses for a given condition. ^*^Significant time × condition interaction, *p* < 0.05.

In the current analysis, plasma β-OHB increased from 0.22 ± 0.07 mM at fasting baseline (pre-OGTT) to 3.7 ± 0.95 mM at 120 min post-OGTT (*p* < 0.001) in the KME condition. The corresponding AUC for the 120 min OGTT period for β-OHB was 418.5 ± 100.2 mM × 120 min in KME compared to 23.8 ± 5.1 mM × 120 min in the placebo condition (*p* < 0.001). The peak β-OHB concentration for OB was 3.7 ± 0.9 mM and occurred at 120 min (see Myette-Côté et al., 2019). There was a significant time x condition interaction (*p* = 0.0367) for the BDNF response to KME ingestion with OGTT in OB (Figure 2). In the placebo condition, plasma BDNF remained unchanged post-OGTT compared to pre-OGTT (143.9 ± 161.9 pg/ml vs. 126.4 ± 134.0 pg/ml, *p* = 0.891). There was a tendency towards a statistically significant increase in plasma BDNF post-OGTT compared to pre-OGTT in the KME condition (188.9 ± 138.2 pg/ml vs. 122.6 ± 129.3 pg/ml; *p* = 0.080).

### Fasting Plasma BDNF Comparison

**Figure 3:**
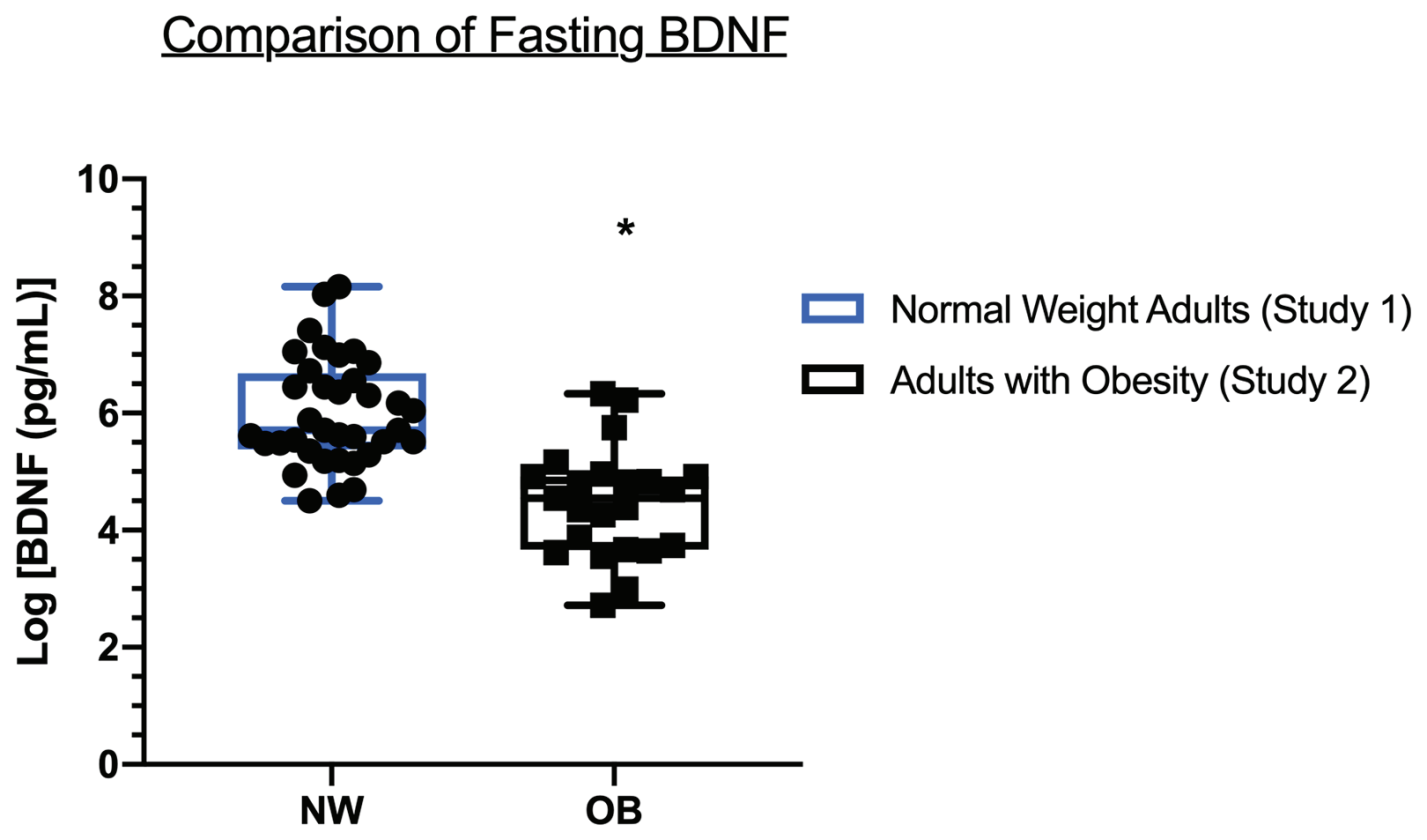
Comparison of fasting plasma BDNF between NW (Study 1) and OB (Study 2). BDNF data are the average of two fasting measures made at baseline (pre-OGTT) of each condition. Data are log-transformed and presented as mean [max, min] with individual data points. NW = normal weight adults, OB = adults with obesity. ^*^Significantly different compared to NW, *p* < 0.05.

Figure 3 demonstrates that fasting levels of plasma BDNF were significantly lower in OB compared to NW (132.8 ± 142.8 pg/ml vs. 639.9 ± 746.8 pg/ml, Hedges *g* = 0.845, *p* = 0.002).

### Exploratory Correlations Between BDNF and Metabolic Outcomes

**Figure 4:**
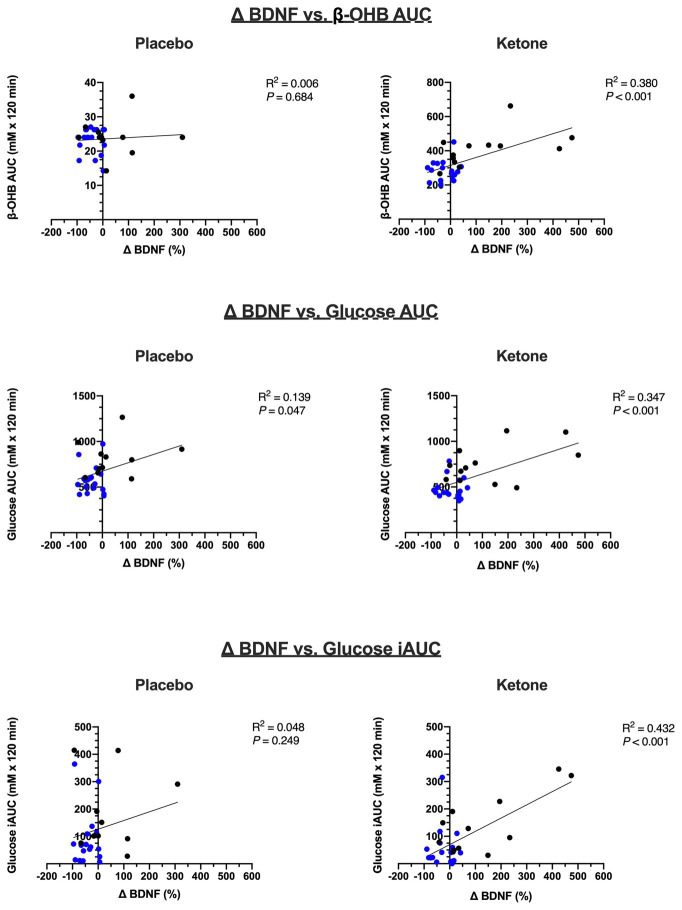
Exploratory correlations of change (∆) in %BDNF, β-OHB, and glucose using combined data from Study 1 and 2 (*n* = 30). Blue symbols are participants from Study 1 (NW; *n* = 18) and black symbols are participants from Study 2 (OB; *n* = 12).

Exploratory correlational analyses were performed to investigate potential relationships between changes in BDNF and metabolic factors in response to OGTT + KME or placebo (Table 2; Figure 4). When assessing each study independently, there was no relationship between BDNF and β-OHB for either NW or OB (Table 2). Interestingly, there was a significant positive strong relationship between percent change in BDNF and glucose iAUC in the KME condition in OB (0.759; *p* = 0.004), whereas no other relationships between BDNF and glucose were apparent in either NW or OB alone.

**Table 2:** Exploratory correlations between change in %BDNF, β-OHB, and glucose.

	Study 1 (NW; *n* = 18)		Study 2 (OB; *n* = 12)	
	Placebo	Ketone	Placebo	Ketone
β-OHB AUC (mM × 120 min)	−0.100 (−0.542, 0.385)	−0.027 (−0.488, 0.445)	0.073 (−0.551, 0.645)	0.456 (−0.159, 0.816)
Glucose AUC (mM × 120 min)	0.005 (−0.462, 0.471)	−0.028 (−0.489, 0.442)	0.191 (−0.462, 0.709)	0.448 (−0.169, 0.813)
Glucose iAUC (mM × 120 min)	−0.039 (−0.497, 0.435)	−0.048 (−0.504, 0.428)	0.085 (−0.543, 0.651)	0.759 (0.328, 0.923)^*^
	Study 1 and 2 Combined (*n* = 30)			
	Placebo		Ketone	
β-OHB AUC (mM × 120 min)	0.078 (−0.296, 0.432)		0.616 (0.329, 0.799)^*^	
Glucose AUC (mM × 120 min)	0.372 (0.007, 0.0650)^*^		0.589 (0.290, 0.783)^*^	
Glucose iAUC (mM × 120 min)	0.221 (−0.159, 0.543)		0.657 (0.389, 0.822)^*^	

Data are: correlation coefficient (95% confidence interval).

* Significant correlation, *p* < 0.05.

When data from Study #1 and #2 were pooled, however, there was a significant positive moderate relationship between β-OHB AUC and percent change in BDNF in the KME condition (*r* = 0.616; *p* < 0.001; Figures 4A,B). There were significant positive moderate relationships between the percent change in BDNF and glucose AUC (*r* = 0.589; *p* < 0.001) and glucose iAUC in the KME condition (*r* = 0.657; *p* < 0.001; Table 2; Figures 4C–F). Interestingly, there was also a significant positive weak relationship between percent change in BDNF and glucose AUC in the placebo condition (*r* = 0.372; *p* = 0.047).

## Discussion

The purpose of this brief research report was to examine the response of plasma BDNF to KME ingestion during an OGTT in NW and OB. The main findings are: (1) in NW, plasma BDNF significantly decreased during an OGTT but this effect was abrogated by KME ingestion; (2) in OB, there was a significant time × condition interaction, such that plasma BDNF during the OGTT remained unchanged with placebo but increased with the KME drink; and (3) plasma BDNF was significantly lower in OB compared to NW.

### The Effect of KME + OGTT on Plasma BDNF

We found that plasma BDNF was significantly lower following an OGTT in NW but not in OB. Mechanistically, hyperglycemia downregulates BDNF release from the brain in lean, normoglycemic men (Krabbe et al., 2007). The reduction in circulating BDNF with hyperglycemia is likely due to changes in cellular release of BDNF as opposed to changes in platelet dynamics, as Araki and colleagues found that plasma, but not serum, BDNF decreases following an OGTT in children with normal and impaired glycemia (Araki et al., 2017).

Similar findings have been reported by Lee et al. (2016) who found that serum BDNF was unchanged following an OGTT in both lean men and men with obesity and metabolic syndrome. However, when expressed as incremental AUC, hyperglycemia appears to reduce circulating BDNF in lean men only. Interestingly, this effect is normalized in men with obesity following a weight loss intervention in proportion to the magnitude of weight lost, suggesting that hyperglycemia differentially affects circulating BDNF based on weight status (Lee et al., 2016).

In the current study, ingestion of a KME prior to OGTT protected against the attenuation of plasma BDNF in NW and increased plasma BDNF in OB. Mechanistically, we are unable to establish the contribution of β-OHB *per se* on BDNF expression given the 11 (Myette-Côté et al., 2019) and 16% (Myette-Côté et al., 2018) attenuation of glucose AUC following OGTT with KME in OB and NW, respectively. Speculatively, the protection of BDNF during OGTT in NW may be due to the glucose attenuation, given that acute hyperglycemia downregulates BDNF release from the brain (Krabbe et al., 2007). As expected, there were marked differences in 2-h incremental glucose AUC between NW and OB in both placebo (88.6 ± 96.7 vs. 193.8 ± 139.5 mM + 120 min, respectively) and KME conditions (52.9 ± 74.2 vs. 142.9 ± 107.9 mM + 120 min, respectively; Myette-Côté et al., 2018, 2019). However, despite these differences, BDNF decreased only in NW placebo condition.

Contrastingly, we observed a strong positive relationship between glucose iAUC and changes in BDNF in the KME condition in OB. This unexpected relationship could suggest that elevated β-OHB augments cellular BDNF release in the presence of elevated glucose. Alternatively, individuals with obesity and poorer glycemic control may benefit the most from elevated β-OHB during an OGTT. Indeed, there was neither a relationship between glucose and BDNF in the placebo condition in OB nor in either condition in NW participants.

Of note, there was a moderate positive relationship between β-OHB AUC and percent change in BDNF when data from NW and OB were pooled. β-OHB has been shown to upregulate BDNF expression in cortical and hippocampal neurons in rodent and *in vitro* models (Marosi et al., 2016; Sleiman et al., 2016; Hu et al., 2018). Seminal mechanistic work by Sleiman et al. (2016) show that exogenous β-OHB stimulates hippocampal BDNF expression by inhibiting histone deacetylase 2 and 3 on the *Bdnf* gene in mice. However, β-OHB administration *in vivo* elicits reductions in circulating glucose, which may interact with mechanisms of direct upregulation of neuronal BDNF by β-OHB.

These preliminary findings require systematic investigation to understand the mechanisms underlying the protection of BDNF by KME during OGTT and whether a KME supplement can increase BDNF under basal conditions. A comprehensive characterization of the temporal profile of BDNF response to β-OHB is required. Extending the observation window beyond 120 min in the basal state may reveal the addition of circulating BDNF from genomic sources, as demonstrated in non-human models (Marosi et al., 2016; Sleiman et al., 2016; Hu et al., 2018). Interestingly, as we were preparing this manuscript, Mujica-Parodi et al. (2020) reported a series of brain imaging studies showing that an acute ketone bolus improves brain network stability compared to acute glucose bolus. These findings have implications for aging and brain-health optimization, as reductions in brain network stability relate to decreased brain activity and lower cognitive function (Mujica-Parodi et al., 2020). However, it has yet to be determined if circulating BDNF is involved in these and other improvements in brain health with KME ingestion.

### Plasma BDNF Is Lower in OB Compared to NW

Here, we found significantly lower levels of basal plasma BDNF in OB compared to NW. Rodent models provide robust evidence for reduced BDNF in obesity (Mainardi et al., 2013; Briana and Malamitsi-Puchner, 2018). BDNF mediates neural plasticity in the arcuate nucleus of the hypothalamus, impacting long-term feeding behavior, such that downregulation of hypothalamic BDNF leads to increased body weight and reduced locomotion in rats (Kernie, 2000; Ozek et al., 2015) – an effect that is reversed by infusion of exogenous BDNF (Kernie, 2000).

The relationship between BDNF and obesity in humans is less clear. Consistent with our findings, Krabbe et al. (2007) and Lommatzsch et al. (2005) found a negative gradient relationship between weight status and plasma BDNF in adults, whereas Golden et al. (2010) reported higher levels of plasma BDNF in women with obesity compared to lean women. Similarly, equivocal findings have been reported for relationships between BDNF and metabolic risk factors. In a cross-sectional analysis, Krabbe et al. (2007) found that plasma BDNF was lower in adults with impaired glycemia (pre-diabetes and type 2 diabetes) compared to those with normal glycemia, regardless of weight status. As well, we have previously reported that serum BDNF is negatively associated with diabetes risk factors in adolescents with obesity (Walsh et al., 2018).

In the current study, a number of factors cofound inference of a direct relationship between weight status and plasma BDNF. OB participants expressed a number of metabolic risk factors, including elevated systolic and diastolic blood pressure, and fasting glucose on the cusp of prediabetes (Table 1; Krabbe et al., 2007). As well, OB were significantly older than NW and plasma BDNF has been shown to be lower with advancing age (Lommatzsch et al., 2005). The observed difference in fasting BDNF between NW and OB in the current study is likely related to overall differences in age and the metabolic milieu, including the possible downregulation of BDNF *via* chronically higher blood glucose (Krabbe et al., 2007; Levinger et al., 2008).

### Limitations

The current study was an exploratory, secondary analysis of blood samples from studies that investigated the glucose-lowering effect of KME ingestion prior to an OGTT. Accordingly, these studies were not designed with plasma BDNF as the primary outcome and a sample size calculation could not be performed. Given that the original studies did not intend to measure BDNF, plasma samples were not subjected to additional centrifuge spin to remove then possible contribution of BDNF from platelets. This represents a possible source of variability in plasma BDNF measures, even with anticoagulant treatment in the plasma tubes. However, potential variability in BDNF due to platelets would be present and consistent across all samples, thereby mitigating potential confounding, especially given that platelet-BDNF dynamics do not appear to change with age (Lommatzsch et al., 2005). However, we did not control for the use of oral contraceptives for female participants. This represents another possible source of variability, as BDNF and platelets fluctuate over the menstrual cycle, and this fluctuation is blunted by oral contraceptives or menopause (Lommatzsch et al., 2005; Pluchino et al., 2009). However, this is unlikely an issue as female participants with a regular menstrual cycle were tested during the follicular phase to control for variability.

The comparison of fasting BDNF between OB and NW is confounded by the significant difference in the age of participants and the unbalanced sex of participants between groups. The original trials from which blood samples were derived were never intended to make inferences from direct comparisons between studies. We opted to include these data as a characterization of the differences between samples but recognize the limitations in drawing direct conclusions about weight status and resting BDNF.

The findings from the current study represent the interaction between β-OHB and OGTT on the BDNF response, which does not allow for the isolation of effect due to β-OHB alone. Future studies should be designed to systematically isolate the effect of β-OHB on circulating BDNF in NW and OB. Despite these limitations, the current study is, to the best of our knowledge, the first to investigate the effect of β-OHB on BDNF in humans and is an important, incremental contribution to the limited available literature.

## Conclusions

In this exploratory research, we confirm that OB have lower fasting BDNF than NW, and that ingestion of a ketone monoester drink results in positive, yet discrepant, changes in plasma BDNF in NW and OB. This response, may, in part, be due to the presence of elevated β-OHB *per se*. To the best of our knowledge, this is the first demonstration of the potential for β-OHB to impact BDNF in humans. Given the potential therapeutic utility of ketone supplements, future studies are needed to elucidate the mechanisms underlying the potential effect of β-OHB on plasma BDNF and whether these changes in circulating BDNF can measurably impact brain-related outcomes. Overall these findings provide preliminary evidence for a potential beneficial interaction between exogenous β-OHB and BDNF and may stimulate broader interest for future studies looking at the utility of KME for protecting metabolic and brain health in people at increased risk.

## Data Availability Statement

The raw data supporting the conclusions of this article will be made available by the authors, without undue reservation.

## Ethics Statement

The studies involving human participants were reviewed and approved by University of British Columbia Clinical Research Ethics Board: ID H16-01846 and ID H16-01846. The participants provided their written informed consent to participate in this study.

## Author Contributions

ÉM-C and JL conceived of the original studies and collected and analyzed all data from which the current, secondary data-analysis was derived. JW conceived of the secondary data analysis and performed all analyses for the current manuscript. JW and ÉM-C wrote the manuscript. All authors revised subsequent versions of the manuscript and approved the final, submitted version.

### Conflict of Interest

JL is the co-Chief Scientific Officer for the not-for-profit Institute for Personalized Therapeutic Nutrition. JL holds shares in Metabolic Insights Inc., a for-profit company developing non-invasive metabolic monitoring devices.

The remaining authors declare that the research was conducted in the absence of any commercial or financial relationships that could be construed as a potential conflict of interest.
